# Fast and accurate haplotype frequency estimation for large haplotype vectors from pooled DNA data

**DOI:** 10.1186/1471-2156-13-94

**Published:** 2012-10-30

**Authors:** Alexandros Iliadis, Dimitris Anastassiou, Xiaodong Wang

**Affiliations:** 1 Center for Computational Biology and Bioinformatics and Department of Electrical Engineering, Columbia University, New York, NY, USA

## Abstract

**Background:**

Typically, the first phase of a genome wide association study (GWAS) includes genotyping across hundreds of individuals and validation of the most significant SNPs. Allelotyping of pooled genomic DNA is a common approach to reduce the overall cost of the study. Knowledge of haplotype structure can provide additional information to single locus analyses. Several methods have been proposed for estimating haplotype frequencies in a population from pooled DNA data.

**Results:**

We introduce a technique for haplotype frequency estimation in a population from pooled DNA samples focusing on datasets containing a small number of individuals per pool (2 or 3 individuals) and a large number of markers. We compare our method with the publicly available state-of-the-art algorithms HIPPO and HAPLOPOOL on datasets of varying number of pools and marker sizes. We demonstrate that our algorithm provides improvements in terms of accuracy and computational time over competing methods for large number of markers while demonstrating comparable performance for smaller marker sizes. Our method is implemented in the "Tree-Based Deterministic Sampling Pool" (TDSPool) package which is available for download at http://www.ee.columbia.edu/~anastas/tdspool.

**Conclusions:**

Using a tree-based determinstic sampling technique we present an algorithm for haplotype frequency estimation from pooled data. Our method demonstrates superior performance in datasets with large number of markers and could be the method of choice for haplotype frequency estimation in such datasets.

## Background

In recent years large genetic association studies involving hundreds or thousands of individuals have become increasingly available, providing opportunities for biological and medical discoveries. In these studies, hundreds of thousands of SNPs are genotyped for the cases and the controls, and discrepancies between the haplotype distributions indicate an association between a genetic region and the disease. Typically, the first phase of a GWAS includes genotyping across hundreds of individuals and validation of the most significant SNPs. One possible approach to reducing the overall cost of GWAS is to replace individual genotyping in phase I with allelotyping of pooled genomic DNA [1-6]. Here, equimolar amounts of DNA are mixed into one sample prior to the amplification and sequencing steps. After genotyping, the frequency of an allele in each position is given [5].

Rather than examining SNPs independent of each other, simultaneously considering the values of multiple SNPs within haplotypes (combinations of alleles at multiple loci in individual chromosomes) can improve the power of detecting associations with disease and is also of general interest with the pooled data. To facilitate haplotype-based association analysis it is necessary to estimate haplotype frequencies from pooled DNA data.

A variety of algorithms have been suggested to estimate haplotype frequencies from pooled data. Available methods fall into two large categories. The first category consists of methods that focus on accurate solutions for small pool sizes (2 or 3 individuals per pool) and considerably large genotype segments. Many well known approaches that focus on small pool sizes use an expectation-maximization (EM) algorithm for maximizing the multinomial likelihood [7-9]. Pirinen et al. [10] extended the gold standard PHASE algorithm [11] to the case of pooled data. They introduced a novel step in the Markov Chain Monte Carlo (MCMC) scheme, during which the haplotypes within each pool were shuffled to simulate individuals on which the original PHASE algorithm could be run to estimate the haplotypes. A method based on perfect phylogeny, HAPLOPOOL, was suggested in [12] and was supplemented with the EM algorithm and linear regression in order to combine haplotype segments. HAPLOPOOL has demonstrated superior performance in terms of accuracy and computational time with respect to the competing EM algorithms. The second category consists of methods that focus on large pools (order of hundred of individuals per pool) and considerably smaller genotype segments. For this scenario, Zhang et al. [13] first proposed a method (PoooL) for estimating haplotype frequencies using a normal approximation for the distribution of pooled allele counts. Imposing a set of linear constraints they transformed the EM algorithm to a constrained maximum entropy problem which they solved using the iterative scaling method. Kuk et al. [14] improved the PoooL methodology, using the ratio of normal densities approximation in the EM, which resulted to the AEM method. Gasbarra et al. [15] introduced a Bayesian haplotype frequency estimation method combining the pooled allele frequency data with prior database knowledge about the set of existing haplotypes in the population. Finally, HIPPO [16] used a multinormal approximation of the likelihood and a reversible-jump Markov chain Monte Carlo (RJMCMC) algorithm to estimate the existing haplotypes in the population and their frequencies. The HIPPO framework is also able to accommodate prior database knowledge for the existing haplotypes in the population and has demonstrated improvements in the performance over the approximate EM - algorithm [16]. In this study we will therefore compare our proposed algorithm with the top performing methods from each category as discussed above, namely HIPPO and HAPLOPOOL.

Naturally, pooling techniques are more prone to errors and offer less possibilities for assessing the quality of the data than individual genotyping. As argued and discussed by Kirkpatrick et al. [12], pooling errors have much greater effect on larger pool sizes as opposed to small pool sizes with respect to the number of incorrect allele calls and the subsequent haplotype estimation. In specific, if σ is the error standard deviation (SD) in the estimates of allele frequencies, 2* σ should be less than the difference between allowable frequency estimates, in order for clustering algorithms to be able to correct the error. As more individuals are included in each pool, the difference between allowable allele frequencies decreases, which results in a higher percentage of incorrect calls. For example in pools of two individuals where the difference between allowable frequency calls is 0.25 (0,0.25, 0.5 ,0.75,1), an accuracy of σ <0.125 will ensure a low rate of incorrect calls (<1%).

In a recent study Kuk et al. [17] examined the efficiency of pooling relative to no pooling using asymptotic statistical theory. They found that under linkage equilibrium (not a typical case!) pooling suffers loss in efficiency when there are more than three independent loci (2^3^ haplotypes) and up to four individuals per pool, whereas accuracy decreases with increasing pool size and number of loci. Rare alleles or linkage disequilibrium (LD) (or both) decrease the number of haplotypes that appear with non-negligible frequencies and thus pooling could remain efficient for larger haplotype blocks. In general, pooling could still remain more efficient in the case where only a small number of haplotypes can occur with appreciable frequency, as also suggested in Barratt et al. [18], and while pool size is kept considerably small.

In this paper we propose a new tree-based deterministic sampling method (TDSPool) for haplotype frequency estimation from pooled DNA data. Our method specifically focuses on small pool sizes and can handle arbitrarily large block sizes. In our study, we examine real data focusing on dense SNP areas, in which only a small number of haplotypes appear with appreciable frequency, so that our scenarios are within the limits of Kuk et al. [17]. We demonstrate that using our methodology we can achieve improved performance over existing state-of-the-art methods in datasets with large number of markers.

## Results

In order to compare the accuracy of frequency estimation between the different methods and under the different scenarios examined, we compared the predicted haplotype frequencies from a given method, *f*, to the gold-standard frequencies, *g*, observed in the actual population. The measure we used was the *χ*^2^ distance between the two distributions which is simply the result of the *χ*^2^ statistic, where *g* is the expected distribution, i.e., *χ*^2^(*f*, *g*) = *Σ*_*i*=1_^*d*^(*f*_*i*_ − *g*_*i*_)^2^/*g*_*i*_ and *d* is the number of gold standard haplotypes [12].

### Datasets

To examine the performance of our methodology we have considered in our experiments real datasets for which estimates of the haplotype frequencies were already available and which cover a variety of dataset sizes.

We have first simulated using the three loci haplotypes and their associated frequencies from the dataset of Jain et al. [19] as the true distribution (Table 1). The haplotypes and their frequencies were estimated using the EM algorithm from a set of 135 individuals genotyped on three SNPs and the estimates were used as the true haplotype distribution. We have simulated datasets with a variable number of pools *T* = 50, 75, 100 and 150. In each pool each individual was randomly selecting a pair of haplotypes according to the distribution of haplotypes. We have created pools with two different pool sizes, 2 and 3 individuals per pool. For each number of pools and each pool size we have created 100 datasets that were used as the datasets for our simulation.

**Table 1:** Haplotypes and their estimated frequencies for the 3 loci dataset

**Haplotype**	**Frequency**
1 0 0	0.082
0 0 1	0.525
1 0 1	0.283
1 1 1	0.106

Next, we considered two more cases with larger number of loci. In the second case which has *L* = 10 loci, we generated data according to the haplotype frequencies of the AGT gene considered in Yang et al. [9]. The haplotypes and their respective frequencies are given in Table 2. The procedure for creating datasets and pools was identical to the three loci case.

**Table 2:** Haplotypes and their estimated frequencies for the 10 loci dataset

**Haplotype**	**Frequency**
1 1 1 1 0 1 1 0 0 0	0.033
1 1 0 1 0 1 1 1 1 0	0.016
1 1 0 1 0 0 1 0 0 1	0.017
1 0 0 1 0 1 1 0 0 1	0.017
1 1 0 1 0 1 1 0 0 1	0.017
1 1 1 1 0 1 1 1 0 1	0.507
0 1 0 1 1 0 0 1 1 1	0.017
1 1 0 0 0 0 1 1 1 1	0.033
0 1 0 1 0 0 1 1 1 1	0.1
1 1 0 1 0 1 1 1 1 1	0.193
1 1 1 1 1 1 1 1 1 1	0.05

The third dataset consisted of SNPs from the first 7Mb (742 kb to 7124.8 kb) of the HapMap CEU population (HapMap 3 release 2- Phasing data). This chromosomal region was partitioned based on physical distance into disjoint blocks of 15 kb. The resulting blocks had a varying number of markers ranging from 2–28. For our purposes we have considered only the datasets that had more than 10 SNPs and less than 20 (which was the maximum number of loci so that HAPLOPOOL could produce estimates within a reasonable amount of time) which resulted in selecting a total of 80 blocks. On each block the parental haplotypes and their estimated frequencies were used as the true haplotype distribution. As in the previous cases, in each block two different pool sizes, 2 and 3 individuals per pool, were considered and four different number of pools per dataset.

### Frequency estimation

**Figure 1:**
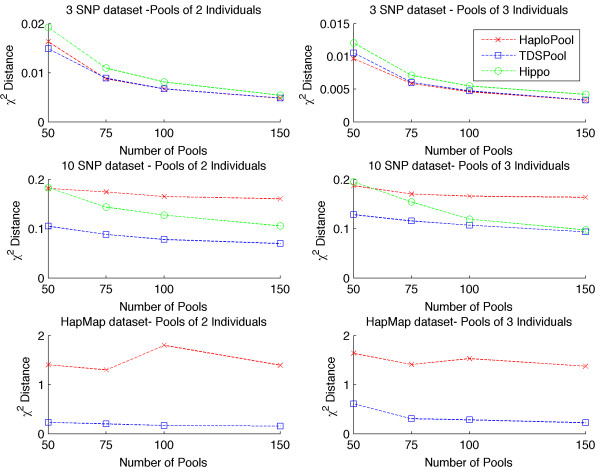
**Accuracy of haplotype frequency estimates.** Estimating χ^2^ distance for 3 loci, 10 loci and HapMap dataset for 50,75, 100 and 150 pools with HAPLOPOOL, TDSPool and HIPPO.

We have examined the accuracy of our method and compared it against HIPPO and HAPLOPOOL on the three datasets described in our previous subsection. In all experiments considered in this subsection the DNA pools were simulated assuming no missing data or measurement error. The performance of the methods is shown in Figure 1.

For the 3 and 10 loci datasets the result presented is the average χ^2^ distance from a 100 simulation experiments, whereas in the HapMap dataset the result presented is the average χ^2^ distance on the 80 datasets considered. For the 3 loci dataset it can be seen that TDSPool and HAPLOPOOL produced similar accuracy. For the remaining two datasets with larger number of loci TDSPool demonstrated superior performance. For the HapMap dataset only TDSPool and HAPLOPOOL were evaluated since the maximum number of loci HIPPO can handle without prior knowledge of the major haplotypes in the population is 10. At the same time even though HAPLOPOOL can in principle handle larger datasets, due to excessive computational time for datasets with 24 and 28 loci we restricted our comparisons to datasets between 10 and 20 loci. We note here as well that since HIPPO is based on a central limit theorem it is likely to be a better approximation in large pools as opposed to small ones that we focus in our study.

From our experiments we can also see that the number of pools also affected accuracy. All algorithms demonstrated improved performance with increasing number of pools in the dataset.

### Noise and missing data

In the previous subsection we have evaluated the performance of our method by simulating DNA pools without missing data and measurement errors. However, in allelotyping pooled DNA, allele frequencies may not be estimated properly in some practical situations and the data are consequently missing or have measurement errors.

**Figure 2:**
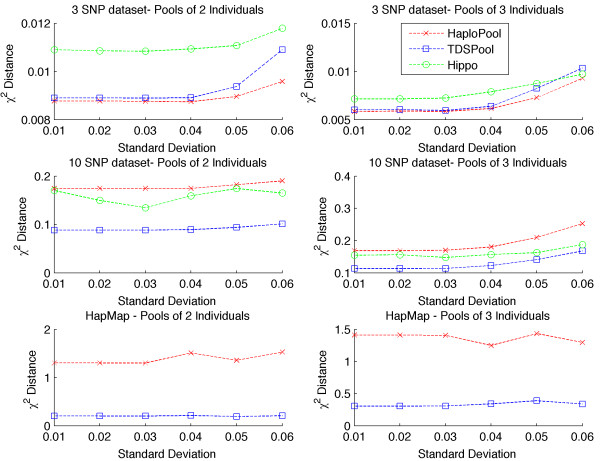
**Accuracy of haplotype frequency estimates with genotyping errors.** Estimating χ^2^ distance for 3 loci, 10 loci and HapMap datasets when noise is added on the pooled allele frequencies.

In order to measure the effect of genotype error on the accuracy of the haplotype frequency estimation and evaluate the performance of our method under such scenarios, we have simulated genotyping error by adding a Gaussian error with SD σ to each called allele frequency. Suppose we denote the correct allele frequency at SNP *j* in pool *i* as c_ij_. The perturbed allele frequency is given by $\hat{c_{\mathrm{ij}}}=c_{\mathrm{ij}}+x$ where *x* ∼ *N*(0, *σ*^2^). After simulating these perturbed haplotype frequencies, we discretize the resulting frequencies to produce perturbed allele counts that are consistent with the number of haplotypes in each pool. We have considered a variety of values for σ, ranging from 0 to 0.06 similar to Kirkpatrik et al. [12]. The perturbed datasets examined were derived from the unperturbed datasets used in the previous subsection with the procedure described above. The results are shown in Figure 2. Due to space limitations we give the results only when the number of pools is 75 but the shape of the figures is similar for the remaining number of pools examined in our previous subsection.

For small number of loci, HAPLOPOOL achieves the best performance. However, for larger datasets TDSPool outperforms all competing methods.

**Figure 3:**
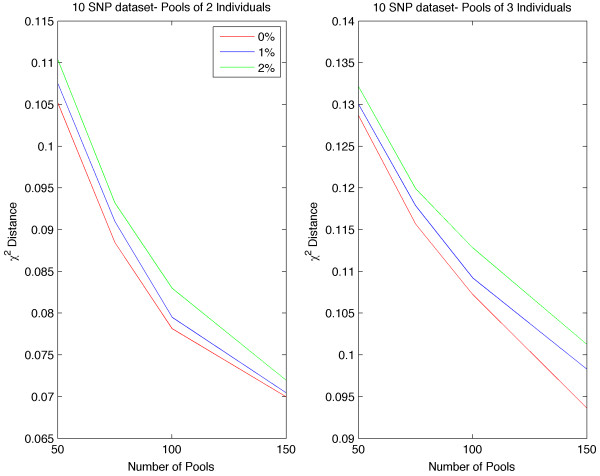
**Accuracy of haplotype frequency estimates with missing data.** Estimating χ^2^ distance for 10 loci dataset with 0,1 and 2% of missing SNPs.

Furthermore, we have evaluated the performance of our methodology using missing data. We have randomly masked 1 and 2% of the SNPs respectively on the 10 loci datasets and estimated the accuracy. As shown in Figure 3, missing SNPs result in small loses in the accuracy and as expected the error decreases with increasing pool number.

### Timing results

The computational times for all datasets are displayed in Table 3. All methods were run with their default parameters. Specifically, for HIPPO the default number of iterations was 100000 and for TDSPool the default number of streams (as will be defined in the "Methods" section) used throughout our experiments was chosen to be 50. Based on these results HIPPO was the slowest performing method in all datasets performing more than 20 times slower than the remaining two algorithms in the ten loci dataset. For the three loci dataset all methods were able to estimate the haplotype frequencies within six seconds. For the ten loci dataset HAPLOPOOL and TDSPool were still able to produce the results in less than three seconds whereas HIPPO demanded more than 58 seconds to finish. For the HapMap datasets again both methods TDSPool and HAPLOPOOL were able to finish the procedure within four seconds. In the ten loci and HapMap datasets TDSPool demonstrated better performance compared to HAPLOPOOL when the number of pools in each dataset was more than 75. Therefore, for all practical applications all methods are fast enough and within limits for researchers to use.

**Table 3:** Timing results

			**Number of pools**		
		**50**	**75**	**100**	**150**
3-loci Dataset					
	TDSPool	0.4458	0.4331	0.4743	0.4861
		0.4260	0.4772	0.5346	0.5350
	HaploPool	0.0697	0.0642	0.0607	0.0674
		0.0593	0.0681	0.0607	0.0691
	HIPPO	2.3593	3.0793	3.8856	5.3911
		2.4182	3.2047	4.1161	5.5873
10-loci Dataset					
	TDSPool	0.8094	0.7778	1.0367	1.1259
		1.0269	1.0805	1.1804	1.3920
	HaploPool	0.5136	0.7381	0.9554	1.4012
		0.8531	1.2331	1.6247	2.4078
	HIPPO	59.5605	62.7163	64.1563	71.0505
		58.8816	64.6515	64.5386	73.9019
HapMap					
Dataset	TDSPool	1.0189	1.1660	1.1765	1.5455
		1.8760	2.0830	2.1848	3.2719
	HaploPool	0.6737	0.9577	1.2679	1.8489
		1.1636	1.6928	2.2006	3.2905

For each dataset in each algorithm the first line corresponds to the case that each pool has 2 individuals whereas the second line to the case that each pool has three individuals. Time is given in seconds.

## Discussion

We have introduced a new algorithm for estimating haplotype frequencies from datasets with pooled DNA samples and we have compared it with existing available packages. We have shown that for datasets with small number of loci our algorithm has comparable performance to state-of-the-art methods in terms of accuracy and computational time but it demonstrates superior performance for datasets with larger number of loci.

Our method specifically focuses on small pool sizes and we have demonstrated the performance on pools of two or three individuals. In our experiments we have partitioned pooled genotype vectors in blocks of 4 SNPs as described in the "Partition-Ligation" subsection. We have chosen to partition the pooled genotypes every 4 SNPs so that computations are performed fast and we avoid cases with huge number of solutions. Partitioning the dataset every 3 SNPs had negligible impact on the accuracy of our results (results not shown) whereas partitioning every 5 SNPs in general can produce block pool genotypes with thousands of solutions, especially when missing data occur.

In the framework developed by Pirinen [16], which had resulted in HIPPO, the algorithm was able to accommodate prior database information on existing haplotypes in a population. Similarly, our methodology offers a framework that can easily incorporate prior knowledge in the form of known haplotypes from the same population as that from which the target pools were created. When such existing haplotypes are known (such as those available from the HapMap), they can be easily introduced in the form of a prior for the counts in the TDSPool algorithm. The presence of the extra information will improve the frequency estimation accuracy in the target population.

## Conclusions

We have introduced a new algorithm for estimating haplotype frequencies from pooled DNA samples using a Tree-Based Deterministic sampling scheme. Algorithms for haplotype frequency estimation from pooled data fall into two categories. The first category consists of algorithms that focus on accurate solutions and allow for considerably large genotype segments and the second category of algorithms that focus on small segments but allow for a large number of individuals per pool. We have compared our methodology with state-of-the-art algorithms from each category, namely HAPLOPOOL and HIPPO. We have focused on scenarios and datasets in which the use of pooling data is suggested for haplotype frequency estimation according to the study of Kuk et al. [17]. In specific, our method focuses on scenarios where pools contain 2 or 3 individuals and we have shown that for such scenarios our method demonstrates comparable or better performance compared with competing algorithms for a small number of loci and outperforms these algorithms for a large number of loci. Furthermore, our TDSPool methodology provides a straightforward framework for incorporating prior database knowledge into the haplotype frequency estimation.

## Methods

In the beginning of the section we introduce some notation. We then present the prior and posterior distribution given the data and derive the state update equations for the TDSPool estimator. We further present the modified partition-ligation procedure adjusted for the pooled data so that we are able to handle larger haplotype vectors and we finally give a summary of the proposed procedure.

### Definitions and notation

Suppose we are given a set of pooled DNA measurements on *L* diallelic loci. We denote the two alleles at each locus by 0 and 1, for convenience of our representation. Following the common notation, we use the counts of allele 1 as the measurement for each allele on each pooled DNA sample, which can be converted from the estimated allele frequencies and consists the pool genotype. Therefore if the size of a pool is *N* individuals, the counts for each allele can vary between 0 and *2N*.

**Figure 4:**
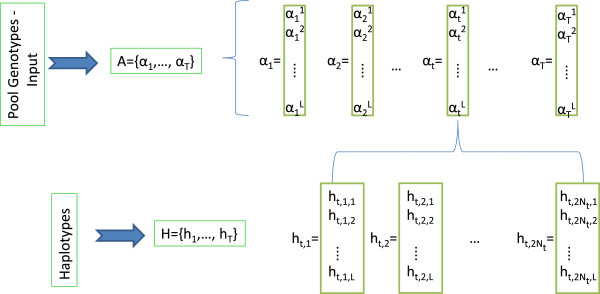
**Schematic representation of the notation used in our methodology.** For each pool genotype (*α*_*t*_) and at each locus, the value of the pool genotype at that locus *α*_*t*_^*j*^ is the sum of the values on that loci across all haplotypes in that pool i.e. $\alpha_{t}^{j}=\Sigma_{i=1}^{2N_{t}}h_{t,i,j}$.

Suppose that we have *T* such pools each one of them with size *N*_*j*_*j* = 1, …, *T*. We denote *α*_*t*_ = {*α*_*t*_^1^, …*α*_*t*_^*L*^} to be the pool genotype of the *t*-th pool where *α*_*j*_^*i*^ ∈ {0, …, 2*N*_*t*_}. Suppose also that *A*_*t*_ = {*a*_1_, …, *α*_*t*_} is a set of pool genotypes of pools up to and including pool *t* and let A denote the full set of pool genotypes. In pool *t* we denote the haplotypes occurring in that pool as *h*_*t*_ = {*h*_*t*,1_, …, *h*_*t*,2*Nt*_} where *h*_*t*,*i*_ ∈ {0, 1}^*L*^ is a binary string of length *L* and the minor allele is present in position *j* in haplotype *i* if *h*_*t*,*i*,*j*_ = 0. We further define *H*_*t*_ = {*h*_1_, …, *h*_*t*_}, similarly to *A*_*t*_ as the set of haplotypes for each genotype pool up to and including pool *t*. A schematic representation of the dataset and the notation used is given in Figure 4.

Let us also define *Z* = {*z*_1_, …*z*_*M*_} , where *z*_*m*_ ∈ {0, 1}^*L*^ is a binary string of length *L* in which 0 and 1 correspond to the two alleles at each locus, as the set containing all haplotype vectors of length *L* that are consistent with any pool genotype in the set *A*. To obtain *Z* from the given dataset *A*, we first enumerate for each *α*_*i*_ the subset *ψ*_*i*_ = {*h*_*i*_^1^, …, *h*_*i*_^*Y*^} *i = 1,…,T* that contains all possible haplotype assignments which are consistent with *α*_*i*_. The set *Z* is then given simply by *Z* = ∪ _*i*=1_^*T*^*ψ*_*i*_ . A set of population haplotype frequencies *θ* = {*θ*_1_, …, *θ*_*M*_} is also associated with the set *Z* of all possible haplotype vectors, where *θ*_*m*_ is the probability with which the haplotype *z*_*m*_ occurs in the total population.

### Probabilistic model

Assuming random mating in the population it is clear that the number of each unique haplotype in *H* is drawn from a multinomial distribution based on the haplotype frequency *θ*[20]. This leads us to the use of the Dirichlet distribution as the prior distribution for *θ*[21] so that *θ* ∼ *D*(*ρ*_1_, …, *ρ*_*M*_)

With mean $E\left\{\theta_{i}\right\}=\frac{\rho_{i}}{\sum_{j=1}^{M}\rho_{\text{j}}}$

Before we calculate the posterior distribution for *θ* we note here that

$$\begin{array}{l} p\left(a_{t}|h_{t}=\left(h_{t,1},\ldots ,ht{{,}_{2N}}_{t}\right)\right)\\ \;=\left\{\begin{array}{l} 1\;\text{if}\;a_{t}\;\text{and}\;h_{t}\;\text{are}\;\text{consistent}\\ \;0\;\text{otherwise} \end{array}\;\right\} \end{array}$$

and similarly

$$p\left(A_{t}|H_{t}\right)=\{1\;if\;A_{t}\;\text{and}\;H_{t}\;\mathrm{are}\;\text{consistent}\;0\;\text{otherwise}$$

Calculating the posterior distribution for *θ* we have:

(1) $$p(\theta |A_{t},H_{t},Z)\propto p(\alpha_{t}|h_{t}=\left(h_{t,1},\ldots ,h_{t,2N_{t}}\right),\theta ,A_{t-1},H_{t-1})p(h_{t}=(h_{t,1},\ldots ,h_{t,2N_{t}})|\theta ,A_{t-1},H_{t-1},Z)p(\theta |A_{t-1},H_{t-1})\propto p(h_{t}=(h_{t,1},\ldots ,h_{t,2N_{t}})|\theta ,Z)p(\theta |A_{t-1},H_{t-1},Z)\propto \prod_{i=1}^{2N_{t}}\theta_{h_{t,i}}\prod_{m=1}^{M}\theta_{m}^{\rho_{m}\left(t-1\right)-1}\propto \prod_{m=1}^{M}\theta_{m}^{\rho_{m}\left(t-1\right)-1+\sum_{i=1}^{2N_{t}}I\left(z_{m}-h_{t,i}\right)}\propto D\left(\rho_{1},(,t,-,1,),+,\sum_{i=1}^{2N_{t}},I(z_{1}-h_{t,i}),,,\ldots ,,,\rho_{M},(,t,-,1,),+,\sum_{i=1}^{2N_{t}},I(z_{M}-h_{t,i})\right)$$

where we denote *ρ*_*m*_(*t*) *m = 1,…,M* as the parameters of the distribution of *θ* after the *t-th* pool and I (*z*_*m*_ − *h*_*t,i*_) with *i = 1,…,2N*_*t*_ is the indicator function which equals 1 when *z*_*m*_ − *h*_*t*,*i*_ is a vector of zeros, and 0 otherwise.

We have shown that the posterior distribution for *θ* is also Dirichlet with parameters as given in (1) and depends only on the sufficient statistics, *T*_*t*_ = {*ρ*_*m*_(*t*), 1 ≤ *m* ≤ *M*} which can be easily updated based on *T*_*t*−1_, *h*_*t*_, *α*_*t*_ as given by (1) i.e. *T*_*t*_ = *T*_*t*_(*T*_*t*−1_, *h*_*t*_, *α*_*t*_).

### Inference problem

Following the notation we used in our previous subsections we can summarize the frequency estimation problem as follows: Given *A* = {*α*_1_, …, *α*_*T*_} the set of observed pool genotype vectors and *Z* = {*z*_1_, …, *z*_*M*_} the set of haplotypes compatible to the pool genotypes in A we wish to infer *H* = {*h*_1_, …, *h*_*T*_} the unknown haplotypes in each pool and *θ* = {*θ*_1_, …, *θ*_*M*_} the haplotype frequencies of all the haplotypes occurring in the population.

### Computational algorithm (TDSPool)

Similar to traditional Sequential Monte Carlo (SMC) methods, we assume that by the time we have processed pool genotype *α*_*t-1*_ we have K sets of solution streams (i.e. sets of candidate haplotypes for pools 1,…, t-1) and their associated weights $\left\{\left(H_{t-1}^{\left(k\right)}|w_{t-1}^{\left(k\right)}\right),k=1,\ldots ,K\right\}$ properly weighted with respect to the posterior distribution *p*(*H*_*t*−1_|*A*_*t*−1_).

Given the set of solution streams and the associated weights we approximate the distribution *p*(*H*_*t*−1_|*A*_*t*−1_) as follows:

(2) $$\hat{p}\left(H_{t-1}|A_{t-1}\right)=\frac{1}{W_{t-1}}\sum_{k=1}^{K}w_{t-1}^{\left(k\right)}I\left(H_{t-1}-H_{t-1}^{\left(k\right)}\right)$$

where $W_{t-1}=\sum_{k=1}^{K}w_{t-1}^{\left(k\right)}\text{,}$and I (●) is the indicator function such that I (*x*-*y*)=1 for *x = y* and I (*x*-*y*) = 0 otherwise.

When we process the pool genotype *t* we would like to make an online inference of the haplotypes *H*_*t*_ based on the pool genotypes *A*_*t*_. Let us further assume that there are *K*^*ext*^ possible haplotype solutions compatible with the genotype of the *t-th* pool, i.e., *h*_*t*_^*i*^, *i* = 1, …, *K*^*ext*^ .

Before we move to the derivation of the state update equation we note here that in the following we will use the fact that for the unknown parameters θ, as we have shown in "Probabilistic Model" subsection, under certain assumptions the prior and posterior distribution are Dirichlet and depend only on a set of sufficient statistics *T*_*t*_ = *T*_*t*_(*T*_*t*−1_, *h*_*t*_, *α*_*t*_)

Therefore, from Bayes’ theorem we have:

(3) $$p(H_{t}|A_{t},Z)\propto p(\alpha_{t}|H_{t},A_{t-1})p(h_{t}|H_{t-1},A_{t-1},Z)p(H_{t-1}|A_{t-1},Z)\propto p(H_{t-1}|A_{t-1},Z)\int p\left(\alpha_{t}|h_{t},\theta \right)p(\theta |h_{t},H_{t-1},A_{t-1},Z)d\theta \int p\left(h_{t}|H_{t-1},\theta ,Z\right)p(\theta |T_{t-1},Z)d\theta \propto p(H_{t-1}|A_{t-1},Z)\int p\left(h_{t}|H_{t-1},\theta ,Z\right)p(\theta |T_{t-1},Z)d\theta \propto p(H_{t-1}|A_{t-1},Z)\int \left(\prod_{i=1}^{2N_{t}}\theta_{h_{t,i}})p(\theta \right|T_{t-1},Z)d\theta \propto p(H_{t-1}|A_{t-1},Z)E_{\theta |T_{t-1}}\{\prod_{i=1}^{2N_{t}}\theta_{h_{t,i}}\}\propto p(H_{t-1}|A_{t-1},Z)[\prod_{i=1}^{2N_{t}}\rho_{h_{t,i}}\left(t-1\right)/{\left(\sum_{m=1}^{M}\rho_{m}\left(t-1\right)\right)}^{2N_{t}}]$$

where $\rho_{h_{t,i}}\left(t-1\right)=\left\{\rho_{z_{m}}\left(t-1\right):h_{t,i}=z_{m}\right\}$

Assuming that we have approximated *p*(*H*_*t*−1_|*A*_*t*−1_) as in (2), we can approximate *p*(*H*_*t*_|*A*_*t*_) using (3) as${\hat{p}}^{\mathrm{ext}}\left(H_{t}|A_{t}\right)=\frac{1}{W_{t}^{\mathrm{ext}}}\sum_{k=1}^{K}\sum_{i=1}^{Kext}w_{t}^{\left(k,i\right)}I\left(H_{t}-\left[H_{t-1}^{\left(k\right)},\left(h_{t,1}^{i},\ldots ,h_{t,2N_{t}}^{i}\right)\right]\right)$_._

The weight update formula is given by

(4) $$w_{t}^{\left(k,i\right)}\propto w_{t-1}^{\left(k\right)}\frac{\prod_{j=1}^{2N_{t}}\rho_{{h^{i}}_{t,j}}^{\left(k\right)}\left(t-1\right)}{{\left(\sum_{m=1}^{M}\rho_{m}^{\left(k\right)}\left(t-1\right)\right)}^{2N_{t}}}$$

### Partition-Ligation

In the partition phase the dataset is divided into small segments of consecutive loci. Once the blocks are phased, they are ligated together using a modified extension of the Partition-Ligation (PL) method [21] for the case of pooled data.

In our current implementation to be able to derive all possible solution combinations for each pool genotype efficiently we have decided to keep the maximum block length to 4 SNPs. Clearly the more SNPs are included in a block the more information about the LD patterns we can capture but at the same time the number of possible combinations increases and becomes prohibitive for more than 5 SNPs. For our experiments in a dataset with L loci we have considered *L/4* blocks of 4 consecutive loci and the remaining SNPs were treated as a separate block.

The result of phasing for each block is a set of haplotype solutions for each pool genotype. Two neighbouring blocks are ligated by creating merged solutions for each pool genotype from all combinations of the block solutions, one from each block. When creating a merged solution for a pool genotype from the two separate solutions (one from each block), since we do not know which haplotypes belong to the same chromosome, all different possible assignments are examined. The TDSPool algorithm is then repeated in the same manner as it was for the individual blocks.

Furthermore, the order in which the individual blocks are ligated is not predetermined. We first ligate the blocks that would produce in each step the minimum entropy ligation. This procedure allows us to ligate first the most homogeneous blocks so that we have more certainty in the solutions that we produce while moving in the ligation procedure.

### Summary of the proposed algorithm

#### Routine 1

● Set the current number of streams m = 1. Define K as the maximum number of streams allowed. Define *H*_0_^1^ ={}.

● For *t = 1, 2,…*

○ Find the *K*^*ext*^ possible haplotype configurations compatible with the pool genotype of the *t-th* pool.

○ For *k = 1,2,…, m , j = 1,…,K*^*ext*^

▪ Enumerate all possible particle extensions $H_{t}^{\left(k,j\right)}=\left[H_{t-1}^{\left(k\right)},\left(h_{t,1}^{j},\ldots ,h_{t,2N_{t}}^{j}\right)\right]$

▪*j* compute the weights *w*_*t*_^(*k*,*j*)^ according to (4)

○ Select and preserve *M = min (K, m· K*^*ext*^*)* distinct sample streams {*H*_*t*_^(*k*)^, *k = 1,…,M*} with the highest importance weights {*w*_*t*_^(*k*)^, *k = 1,…,M*} from the set {*H*_*t*_^(*k*,*j*)^, *w*_*t*_^(*k*,*j*)^, *k = 1,…,m, j = 1,…, K*^*ext*^ }

○ Update the number of counts of each encountered haplotype in each stream

○ Set *m = M*

#### TDSPool ALGORITHM

● Partition the genotype dataset *G* into *B* subsets.

● For *b = 1,…,B ,* apply Routine 1 so that all segments are phased and for each one keep all the solutions contained in the top *K* particles.

● Until all blocks are ligated, repeat the following

○ Find the blocks that if ligated would produce the minimum entropy

○ Ligate the blocks, following the procedure described in the Partition-Ligation section

## Authors’ contributions

All authors contributed equally to this work. All authors read and approved the final manuscript.
